# Distribution of TENORM-contaminated soils in residential areas of Bangka Island, Indonesia

**DOI:** 10.1371/journal.pone.0348187

**Published:** 2026-10-05

**Authors:** Yo Ishigaki, Katsumi Shozugawa, Ryo Ichimiya, Harrizki Arie Pradana, Sidik Permana, Kan Shimazaki, Kazunori Hayashi, Adi Rahmansyah Amir Abdullah, Cici Wulandari

**Affiliations:** 1 Research Center for Realizing Sustainable Societies, University of Electro-Communications, Tokyo, Japan; 2 Graduate School of Arts and Sciences, The University of Tokyo, Tokyo, Japan; 3 Department of Informatics Engineering, Faculty of Information Technology, ISB Atma Luhur, Kota Pangkal Pinang, Bangka Belitung, Indonesia; 4 Physics Department, Nuclear Science and Engineering Department, Institut Teknologi Bandung, Bandung, Jawa Barat, Indonesia; 5 Faculty of Biology-Oriented Science and Technology, Kindai University, Kinokawa, Japan; Universiti Teknologi Malaysia, MALAYSIA

## Abstract

Radioactive contamination in residential areas of Bangka Island, Indonesia, a major tin- and rare earth-producing region, has rarely been studied, although material stored near mineral-processing workshops may pose a long-term health risk. We collected 46 soil samples around 9 mineral-processing workshops in the Selindung District, Pangkal Pinang City: residential soils from 20 locations at two depths (*n* = 40) and 6 workshop-related sediments. High-purity germanium detector analysis showed that 13 of the 40 residential soil samples exceeded the screening criteria of the IAEA Safety Standards Series No. RS-G-1.7 for naturally occurring radioactive material. Residential contamination was highly heterogeneous and was not explained by a simple monotonic distance relationship (Spearman’s *ρ* = −0.12, *p* = 0.61). Two upper outliers occurred at 5 and 10 m from the nearest workshops, including the maximum residential activity of 12,992 Bq/kg detected at 5 m, in areas frequently accessed by local residents. Five of 17 locations with quantifiable paired measurements (including 1 tied pair; *n* = 16 for the Wilcoxon signed-rank test) showed higher activity at 20 cm than at 10 cm, and the paired depth difference was not statistically significant (Wilcoxon *p* = 0.079), indicating an irregular rather than consistently surface-dominated vertical distribution. Screening-level outdoor annual effective doses for the quantifiable residential samples ranged from 0.051 to 8.46 mSv/y (median 0.42 mSv/y), with the maximum obtained 5 m from a workshop. Nuclide composition analysis further revealed that the activity concentrations of the ^238^U and ^232^Th series increased with total radioactivity, whereas ^40^K showed only a weak correlation. Maximum activities of ^238^U and ^232^Th approached levels reported by the IAEA for high-purity monazite sand (6,000–20,000 Bq/kg). These findings demonstrate spatial and vertical heterogeneity of technologically enhanced naturally occurring radioactive material in residential zones, indicating a need for site-specific assessment and long-term monitoring.

## Introduction

Bangka Island, Indonesia, is located at the southern end of the Southeast Asian Tin Belt, which extends from Myanmar to Thailand and the Malay Peninsula to Indonesia [1]. In addition to government and private enterprises, approximately 50,000 small-scale miners operate tin mines on the island. The tailings generated during mining contain various minerals rich in rare earth elements (REEs), including monazite and zircon, with the zirconium content exceeding 5.0 wt.% [2]. The region’s estimated REE mineral resources and zircon reserves amount to 951,000 t, whereas the estimated monazite reserves in Bangka’s coastal areas reach 471,087,689 m^3^ [3]. Thus, Bangka Island is emerging as a critical source of essential minerals for modern high-tech industries, beyond its traditional role in tin production.

The extraction of monazite and zircon from tin tailings through additional processing is a classic example of technologically enhanced naturally occurring radioactive materials (TENORM). High-density minerals containing naturally occurring radioactive materials (NORM), collectively known as heavy mineral sands (HMS), require meticulous radiation safety management for waste generated by mining operations and processing plants. According to International Atomic Energy Agency (IAEA) technical reports, high-purity monazite sand can contain uranium-series radioactivity concentrations ranging from 6,000–20,000 Bq/kg, with a thorium content of approximately 4% by weight. High-purity zircon sand typically exhibits radioactivity concentrations of 4,000 Bq/kg for uranium and 600 Bq/kg for thorium [4]. Industries handling HMS with high radioactivity concentrations should operate under carefully developed and rigorously reviewed waste management plans [5].

Several countries have reported environmental challenges associated with HMS processing. In Nigeria’s Jos Plateau, processing facilities have recorded external radiation dose rates ranging from 10 to 80 μSv/h, with widespread environmental dispersion of residues [6]. In Malaysia, tin mining waste slag has radioactivity concentrations of 1,200–2,000 Bq/kg, necessitating improved disposal site selection and monitoring systems [7]. In Brazil, processing facilities have reported ^238^U concentrations of 2,300 Bq/kg in zircon sand and 6,000–20,000 Bq/kg in monazite sand, raising concerns about soil and groundwater contamination through rainwater transport [8].

Large-scale processing plants on Bangka Island use sophisticated separation techniques, including cone separators, gravity separators, tables, rotary dryers, high-voltage electrostatic separators, and magnetic separators, to isolate non-magnetic zircon and magnetic monazite from crude tin concentrate [9]. However, rudimentary gravity separation methods using inclined shaking tables, water flow, and dryers are also commonly used locally, with ongoing optimization of parameters such as water flow rate, feed rate, and table inclination angle [10]. Small-scale miners on Bangka Island often construct small workshops near rivers or ponds for water-based separation, raising concerns about less stringent management practices than large-scale facilities.

Small-scale mining operations are widespread across Bangka Island (Fig 1), with numerous small workshops located in residential areas of the provincial capital, Pangkal Pinang. These workshops often operate near residential buildings, with refined products and slag typically stored in flexible intermediate bulk containers without adequate measures to prevent dispersion. At a temporary storage area in a workshop, the dose rate near the surface of the stored material reached 19.7 μSv/h (Fig 1d; measured using a TCS-172 scintillation survey meter; Aloka Co., Ltd., Tokyo, Japan), substantially above typical environmental background levels, highlighting the potential radiological significance of these facilities. Additionally, boundaries between workshops and residential areas are often unclear and unrestricted, with children frequently using these areas as playgrounds, posing health risks from external exposure, uranium intake, and radon inhalation. Furthermore, uranium-contaminated soil and groundwater may enter the food chain through agricultural activities, raising concerns about long-term health impacts.

**Fig 1:**
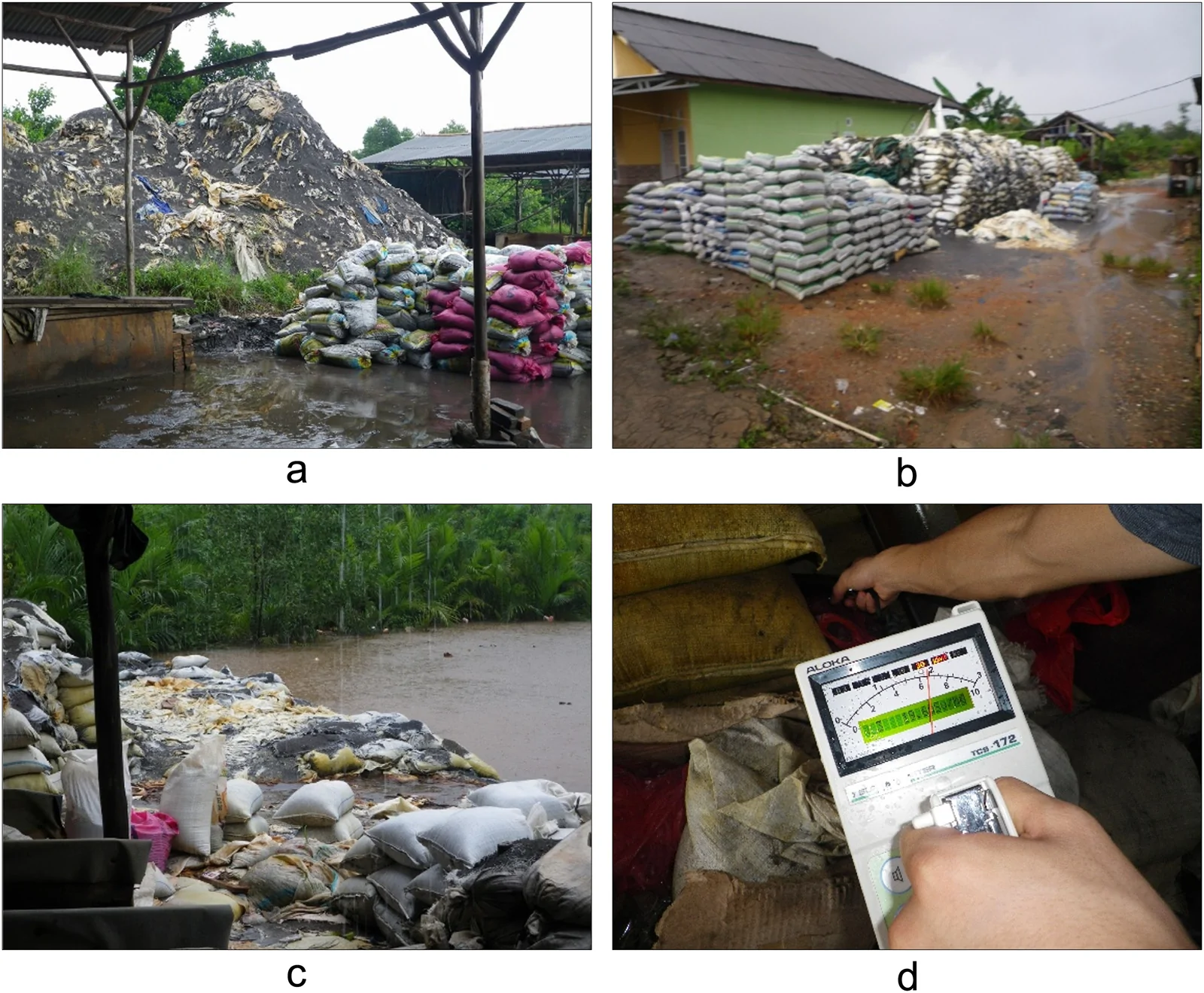
Small-scale mineral-processing workshops on Bangka Island, Indonesia. (a) Processing residues in torn plastic bags. (b) Product storage adjacent to residential buildings. (c) Sediment discharge into a river. (d) Ambient dose equivalent rate measured near the surface of stored material with a TCS-172 scintillation survey meter (Aloka, Tokyo, Japan), reaching 19.7 μSv h^−1^. Photographs were taken by the authors on 22 November 2017, preceding the 2021 sampling campaign, and are published under CC BY 4.0.

Enhanced environmental monitoring, particularly regular soil surveys, is essential for addressing environmental contamination and public exposure concerns. Previous studies on Bangka Island have primarily focused on regional resource evaluations and surface radiation measurements [11,12]. To the best of our knowledge, however, no previous study has separately quantified the ^238^U-series, ^232^Th-series, and ^40^K activity concentrations of residential soils collected at two depths within meters to tens of meters of small-scale mineral-processing workshops. In contrast, the aim of this study was to elucidate the spatial heterogeneity and depth-dependent distribution of radioactive materials in and around small-scale processing workshops scattered throughout the island, based on soil sampling surveys in both workshop sites and adjacent residential areas. This investigation represents a vital step toward balancing the sustainable development of critical minerals with protecting the health of residents.

## Materials and methods

### Study area and site selection

Field surveys were conducted from August 25–30, 2021, in the Selindung District of Pangkal Pinang City, Bangka Belitung Province, Indonesia. The Selindung District is a low-relief urban area on the eastern coast of Bangka Island, traversed by small rivers and drainage channels and characterized by a high concentration of government facilities, residential areas, and educational institutions. Because monazite characteristically incorporates thorium and uranium, the heavy-mineral fraction of the placer-derived mineral sands of Bangka Island is naturally enriched in the ^232^Th and ^238^U decay series. Under the tropical rainfall regime, these rivers and drainage channels can redistribute discharged processing residues into surrounding soils. This district contains an estimated 50–100 mineral-processing workshops. The raw mineral sands processed at these workshops are excavated elsewhere, in open-pit and offshore mining operations, and transported to the district; the workshops are thus processing sites rather than extraction sites.

Site selection was based on comprehensive criteria encompassing geographic, operational, and environmental factors. All workshops included in the study were visible from public roads and accessible for sampling activities.

Operational characteristics of the selected workshops included the processing of rare earth minerals using water-based gravity separation, which exploits the high specific gravities of monazite and zircon [13,14], with raw materials and concentrates visibly stored in bags. These facilities were located within residential areas, with houses situated within 100 m, and characterized by small-scale, non-industrial, and informal operations.

Environmental conditions were also a key consideration in site selection. The selected sites were located near water bodies and exhibited evidence of sediment accumulation and material discharge. Facilities that did not meet these criteria or were inaccessible owing to private property restrictions were excluded from the study. This was an exploratory, risk-oriented survey targeting accessible workshops with visible mineral-processing activities and potential residential contact, rather than a statistically representative survey of all workshops in the district.

### Sample collection

Three types of sediment samples were collected from the study area. Pre- and post-processing sand samples were obtained, with the oral authorization of the site supervisors and workers, from workshops where these materials were stored in bags on the premises. At discharge points where workshop sediments had entered rivers, leaked sandy material was collected. Additionally, surface soil was sampled from selected residential properties located within several tens of meters of each workshop, after obtaining the property owner’s oral permission. For residential sampling, we adopted the EPA definition of surface soil as the upper 15 cm of the soil profile [15] and considered potential soil remediation through construction activities by collecting samples at depths of 10 and 20 cm. Workshop-to-residence distances were determined from GPS coordinates recorded at the time of sampling.

All soil samples were subjected to standardized preparation procedures. Samples were dried at 150°C for 1 h in a laboratory oven and then ground and sieved to produce a uniform grain size. The purpose of oven drying was solely to remove soil moisture prior to gamma spectrometry; the measured gamma-emitting radionuclides are not affected by possible alterations of moisture-sensitive mineral phases at this temperature. The processed samples were sealed in 250-mL polyethylene containers and stored for 32 days to ensure equilibrium in the uranium and thorium decay series below ^222^Rn and ^220^Rn.

Additional information regarding the ethical, cultural, and scientific considerations specific to inclusivity in global research is included in the Supporting Information (S1 Checklist).

### Radioactivity measurements

Radioactivity was measured using a high-purity germanium detector (Model GR2519; Canberra Corp., Toledo, OH, USA) equipped with a crystal detector with a diameter of 55.5 mm, length of 53 mm, 25% relative efficiency, and full-width at half-maximum of 1.9 keV. Gamma spectra were analyzed using PCA II Nucleus software, with a counting time of 40,000 s per sample to minimize counting errors.

The detector was calibrated using IAEA standard sources: RGU-1 for ^238^U, RGTh-1 for ^232^Th, and RGK-1 for ^40^K. Activity concentrations (A) were calculated as follows:


$$\begin{array}{c} A=\frac{N_{net}}{t_{live}\;\varepsilon (E)\;\;P_{\gamma }\;m} \end{array}$$ (1)

where A is the activity concentration (Bq kg^−1^), *N*_net_ is the net full-energy peak area obtained after subtraction of the continuum beneath the region of interest, *t*_live_ is the live time of the measurement (s), *ε*(E) is the full-energy-peak efficiency at gamma energy *E*, *P*_γ_ is the gamma emission probability, and *m* is the sample mass (kg) [16].

For the ^238^U series, activities from ^214^Pb (351.93 keV) and ^214^Bi (609.32 keV) were combined using inverse-variance weighting, with weights defined as 1/σ² based on the 1σ measurement uncertainties [17], assuming secular equilibrium with ^226^Ra in the ^238^U decay chain. For the ^232^Th series, activities derived from ^228^Ac (911.2 keV) and ^208^Tl (2614.5 keV) were combined in the same manner. When the 2614.5 keV line could not be reliably quantified because of low counting statistics and reduced full-energy peak efficiency at high gamma energies, the lower-energy ^208^Tl line at 583.19 keV was used as an auxiliary alternative.

Energy calibration was performed using the certified γ-lines of RGU-1, RGTh-1, and RGK-1 over 186–2,615 keV. The full-energy-peak efficiency was determined from the same three IAEA reference materials measured in the identical container geometry as the samples and fitted as a polynomial in log–log space; because the reference lines span the full energy range of the analyte γ-lines, all analyte-line efficiencies were obtained by interpolation. Because the reference materials share a similar silicate matrix and density with the environmental soil samples, self-attenuation, density effects, and coincidence-summing effects were expected to be reduced by the matched-matrix and matched-geometry calibration.

Net full-energy peak areas were obtained by fitting a Gaussian function to each photopeak and subtracting the continuum beneath the region of interest estimated by the peak-analysis software. A peak was accepted as detected when the fitted net area exceeded three times the standard deviation of the counts in the surrounding channels; otherwise, the radionuclide was reported as not detected (ND). A separate ambient background spectrum was not recorded in this campaign; the sample-specific continuum subtraction intrinsically accounts for the Compton and smooth environmental continuum at each analyte energy. Because the photopeak-to-continuum ratio was large for all samples exceeding the screening criterion, this approach has a negligible effect on the reported activities and is relevant only to the lowest-activity samples, which are reported as ND.

All spectra were corrected for live time. No explicit self-attenuation, density, or coincidence-summing corrections were applied; based on operational experience, the residual coincidence-summing effect contributes an additional uncertainty of approximately 1%. The reported uncertainties represent 1σ counting statistics of the net peak areas propagated through the inverse-variance-weighted averaging; systematic components associated with the efficiency calibration and the certified activities of the reference materials are not included. Absolute activities may therefore include systematic uncertainty, whereas relative comparisons among samples remain robust.

Approximate detection limits were conservatively estimated using a Currie-type approach (*L*_D_ ≈ 4.65*σ*₀) [18], approximating *σ*₀ by the 1σ counting uncertainties of the lowest-activity quantified samples; representative values were approximately 5, 10, and 30 Bq kg^−1^ for the ^238^U series, ^232^Th series, and ^40^K, respectively. Because the principal findings are based on samples with activities two to three orders of magnitude above these detection limits, uncertainty in the detection-limit estimates did not affect the study conclusions.

### Radiological screening assessment

To provide a quantitative screening-level interpretation of the measured activity concentrations, the activity of the ^238^U series derived from ^214^Pb and ^214^Bi was used as a proxy for ^226^Ra activity under the secular-equilibrium assumption described above. For samples with quantifiable U- and Th-series activities, radium equivalent activity (Ra_eq_), absorbed gamma dose rate in air (*D*), external and internal hazard indices (*H*_ex_ and *H*_in_), outdoor annual effective dose (AEDE_out_), and excess lifetime cancer risk (ELCR) were calculated as follows [19–21]:


$$\begin{array}{c} {\text{Ra}}_{\text{eq}}\;=\;A_{\text{Ra}}\;+\;\text{1}.\text{43 }A_{\text{Th}}\;+\;\text{0}.\text{077 }A_{\text{K}} \end{array}$$ (2)

$$\begin{array}{c} D\;=\;\text{0}.\text{462 }A_{\text{Ra}}\;+\;\text{0}.\text{604 }A_{\text{Th}}\;+\;\text{0}.\text{0417 }A_{\text{K}} \end{array}$$ (3)

$$\begin{array}{c} H_{\text{ex}}\;=\;A_{\text{Ra}}/\text{370 }+\;A_{\text{Th}}/\text{259 }+\;A_{\text{K}}/\text{4810} \end{array}$$ (4)

$$\begin{array}{c} H_{in}\;=\;A_{\text{Ra}}/\text{1}\text{8}\text{5}\;+\;A_{\text{Th}}/\text{2}\text{5}\text{9}\;+\;A_{\text{K}}/\text{4}\text{8}\text{1}\text{0} \end{array}$$ (5)

$$\begin{array}{c} {\text{AEDE}}_{\text{out}}\;=\;D\;\times \text{ 8760 }\times \;\text{0}.\text{2}\;\times \;\text{0}.\text{7}\;\times \;{\text{10}}^{-\text{6}} \end{array}$$ (6)

$$\begin{array}{c} \text{ELCR }=\;{\text{AEDE}}_{\text{out}}\;\times \;{\text{10}}^{-\text{3}}\;\times \text{ 70 }\times \;\text{0}.\text{055} \end{array}$$ (7)

where *A*_Ra_, *A*_Th_, and *A*_K_ are the activity concentrations of the ^226^Ra-equivalent U series, ^232^Th series, and ^40^K, respectively, in Bq kg^−1^; *D* is expressed in nGy h^−1^; and AEDE_out_ is expressed in mSv y^−1^. An outdoor occupancy factor of 0.2 and a conversion coefficient of 0.7 Sv Gy^−1^ were adopted [19]. The ELCR calculation assumed a 70-year exposure duration and a nominal cancer detriment coefficient of 0.055 Sv^−1^ [21]. Non-detected ^40^K activities were treated as zero, and samples for which either the U- or Th-series activity could not be quantified were excluded from these calculations. Because these equations assume a spatially uniform radionuclide distribution and generic occupancy conditions, the resulting values were interpreted as screening indices rather than measurements of individual dose or cancer risk. In addition, compliance with the IAEA RS-G-1.7 screening levels [22] was assessed using the sum of the ratios of each activity concentration to its corresponding screening value (1 Bq/g for the ^238^U and ^232^Th series and 10 Bq/g for ^40^K), with a sum ≥ 1 regarded as exceeding the criterion.

### Data analysis

#### Statistical analysis.

The total radioactivity of each sample was calculated as the sum of the activity concentrations of the ^238^U series, ^232^Th series, and ^40^K. Non-detected radionuclide activities were treated as zero when calculating total activity, and observations for which total activity could not be quantified were excluded from the corresponding analysis. Statistical analyses of spatial and depth-dependent patterns were restricted to the residential samples.

To avoid treating the paired depth samples as independent observations in the distance analysis, one representative total activity concentration was assigned to each residential location (*n* = 20), defined as the higher of the values measured at 10 and 20 cm. The monotonic association between distance from the nearest workshop and representative total activity was evaluated using Spearman’s rank correlation.

As a complementary group comparison, the sample median residential distance of 7 m was used as a descriptive threshold. Locations at or below 7 m were classified as the near-range group (*n* = 11), whereas locations farther than 7 m were classified as the far-range group (*n* = 9). Differences in the activity distributions were evaluated using a two-sided Mann–Whitney U test. Equality of dispersion between the two groups was assessed using the Brown–Forsythe test, implemented as a median-centered Levene test. Potential outliers among the 20 residence-level representative values were identified using the 1.5 × interquartile-range criterion.

Depth-dependent differences were evaluated by calculating the difference in total radioactivity (10 − 20 cm) for each Residence ID and applying a two-sided Wilcoxon signed-rank test, excluding zero differences, to the 17 residential locations at which total activity was quantifiable at both depths. All tests were two-sided, with a significance level of 0.05. Given the exploratory sample size, *p*-values were interpreted together with the direction, magnitude, and distribution of the observed values.

No district-wide spatial interpolation was performed because the study was designed as a site-specific field investigation rather than a district-scale geostatistical survey.

#### Nuclide composition trends analysis.

To evaluate compositional trends, the activity concentrations of the ^238^U series, ^232^Th series, and ^40^K were plotted against the total radioactivity of each sample. Linear regression analysis was applied to summarize the relationships between total activity and each nuclide concentration. The coefficient of determination (R²) was used as a descriptive indicator of the strength of these empirical trends.

All statistical analyses and visualizations were conducted in Python with the scipy, numpy, and matplotlib packages.

## Results

Nine mineral-processing workshops were identified in the study area (Fig 2). The spatial distribution of these workshops between the nearest and farthest pairs ranged from 223 to 1,216 m. At two of the nine workshops where access was granted, six samples were collected, including bagged and leaked sediments. Additionally, surface soil samples were collected at depths of 10 and 20 cm from 20 residential locations surrounding the workshops.

**Fig 2:**
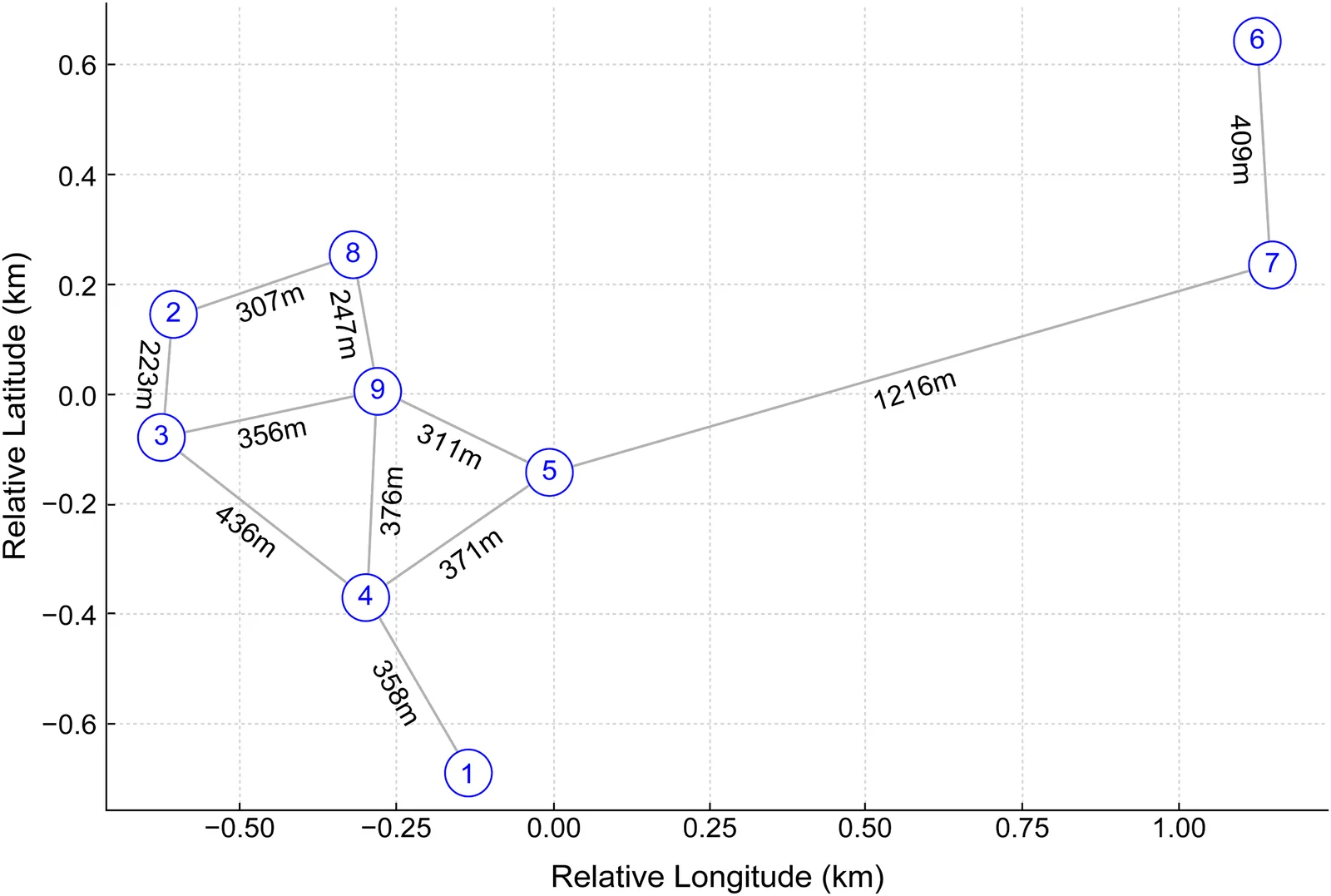
Relative spatial configuration of the nine workshops (IDs 1–9 as in Table 1). Positions are in metres relative to the centroid of the nine workshops; absolute coordinates are withheld to protect residents’ privacy. Gray lines connect workshop pairs and are annotated with the straight-line separation in metres.

**Table 1:** Sampling locations and activity concentrations of the ^238^U series, ^232^Th series, and ^40^K in 46 soil and sediment samples collected around nine mineral-processing workshops, Selindung District, Pangkal Pinang City, Bangka Island, Indonesia, 25–30 August 2021. Samples comprise 6 workshop-related sediments (bagged material stored on the premises; material leaked at river discharge points) and 40 residential soils taken at 10 and 20 cm depth at 20 residential locations. Activity concentrations are in Bq kg^−1^ dry weight; Total is the sum of the three preceding columns.

Sample ID	Workshop ID	Residence ID	Type of Sediment	Distance (m)	Depth (cm)	Radioactivity (Bq/kg)			
						^238^U series	^232^Th series	^40^K	Total
1	1	N/A	Bagged	0	0	134 ± 2	159 ± 2	ND	293
2	2	N/A	Bagged	0	0	2781 ± 8	7324 ± 15	532 ± 21	10636
3	2	N/A	Bagged	0	0	7644 ± 13	9583 ± 17	732 ± 27	17959
4	2	N/A	Bagged	0	0	4591 ± 11	7045 ± 16	569 ± 24	12205
5	2	N/A	Bagged	0	0	1869 ± 9	3973 ± 15	404 ± 22	6246
6	2	N/A	Leaked	0	0	48 ± 2	110 ± 2	ND	158
7	2	1	Residential	5	10	3562 ± 11	8637 ± 19	792 ± 28	12992
8	2	1	Residential	5	20	2395 ± 10	5736 ± 17	448 ± 24	8579
9	2	2	Residential	5	10	351 ± 4	631 ± 6	ND	982
10	2	2	Residential	5	20	114 ± 2	182 ± 3	106 ± 7	403
11	2	3	Residential	4	10	98 ± 2	184 ± 3	ND	282
12	2	3	Residential	4	20	75 ± 2	125 ± 2	ND	200
13	2	4	Residential	5	10	147 ± 2	211 ± 3	ND	357
14	2	4	Residential	5	20	112 ± 2	163 ± 3	ND	275
15	2	5	Residential	50	10	150 ± 2	245 ± 3	101 ± 8	497
16	2	5	Residential	50	20	64 ± 2	85 ± 2	ND	149
17	3	6	Residential	1	10	195 ± 3	419 ± 5	ND	614
18	3	6	Residential	1	20	135 ± 3	270 ± 4	259 ± 11	664
19	3	7	Residential	6	10	254 ± 4	584 ± 6	184 ± 11	1022
20	3	7	Residential	6	20	100 ± 2	204 ± 3	ND	304
21	3	8	Residential	10	10	1373 ± 7	3045 ± 10	265 ± 15	4683
22	3	8	Residential	10	20	1824 ± 7	3728 ± 12	ND	5552
23	3	9	Residential	20	10	1125 ± 7	2139 ± 11	242 ± 17	3507
24	3	9	Residential	20	20	1530 ± 8	3011 ± 12	ND	4542
25	3	10	Residential	4	10	272 ± 3	475 ± 5	ND	746
26	3	10	Residential	4	20	255 ± 3	457 ± 5	314 ± 11	1026
27	4*	11	Residential	40	10	658 ± 6	1488 ± 10	ND	2146
28	4*	11	Residential	40	20	54 ± 2	130 ± 3	ND	184
29	4*	12	Residential	80	10	41 ± 2	58 ± 2	ND	99
30	4*	12	Residential	80	20	22 ± 1	—	ND	—
31	4*	13	Residential	50	10	28 ± 2	47 ± 2	ND	74
32	4*	13	Residential	50	20	—	—	ND	—
33	4*	14	Residential	25	10	57 ± 2	77 ± 3	ND	134
34	4*	14	Residential	25	20	55 ± 2	79 ± 2	ND	134
35	4*	15	Residential	35	10	905 ± 6	944 ± 7	ND	1849
36	4*	15	Residential	35	20	879 ± 6	943 ± 8	ND	1822
37	5	16	Residential	20	10	2357 ± 10	2969 ± 12	ND	5326
38	5	16	Residential	20	20	875 ± 6	1217 ± 8	418 ± 15	2511
39	6	17	Residential	5	10	912 ± 9	2005 ± 14	ND	2917
40	6	17	Residential	5	20	574 ± 6	1132 ± 10	ND	1707
41	7	18	Residential	7	10	37 ± 1	46 ± 2	ND	83
42	7	18	Residential	7	20	19 ± 1	—	60 ± 6	—
43	8	19	Residential	5	10	49 ± 2	71 ± 2	ND	120
44	8	19	Residential	5	20	70 ± 2	112 ± 3	ND	181
45	9	20	Residential	7	10	264 ± 3	434 ± 4	401 ± 12	1099
46	9	20	Residential	7	20	103 ± 2	138 ± 2	ND	241

Series activities are inverse-variance-weighted means of ^214^Pb (351.93 keV) and ^214^Bi (609.32 keV) for the ^238^U series, assuming secular equilibrium with ^226^Ra, and of ^228^Ac (911.2 keV) and ^208^Tl (2614.5 keV, or 583.19 keV where the 2614.5 keV line was not reliably quantified) for the ^232^Th series. Values after ± are 1σ counting uncertainties propagated through the weighting and given as absolute activities (*A* × *σ*_rel_); systematic components are not included.

ND, not detected: net peak area below three times the standard deviation of the surrounding channels. Approximate detection limits (Currie-type, *L*_D_ ≈ 4.65*σ*₀) were 5, 10, and 30 Bq kg^−1^ for the ^238^U series, ^232^Th series, and ^40^K. ND was treated as zero in Total.

—, not quantified: the photopeaks required for the weighted mean gave no acceptable fit, so neither an activity nor an upper bound could be assigned. Unlike ND, this is not an evaluated value below a detection limit. Total is reported as — for Sample IDs 30, 32, and 42, which were excluded from the statistical analyses and from S1 Table. Sample 30 shows all three cases in one row: ^238^U series quantified (22 ± 1 Bq kg^−1^), ^232^Th series not quantified (—), ^40^K below detection (ND), Total —.

*, workshop that had ceased operations within the year before sampling. Distance is to the nearest workshop, from GPS coordinates; 0 m denotes sampling within the workshop premises, and depth 0 cm denotes a surface sample. N/A, not applicable to workshop samples.

Table 1 presents the complete sampling and analysis results. Among the nine workshops, one (Workshop ID 4) had ceased operations within the year before our sampling campaign; however, its environmental impact remained relevant to the study objectives.

The maximum concentrations recorded in Sample ID 3 were 7,644 Bq/kg for ^238^U and 9,583 Bq/kg for ^232^Th, approaching the levels reported by the IAEA for high-purity monazite sand (6,000–20,000 Bq/kg). Furthermore, among the 40 residential soil samples analyzed, 13 (32.5%) exceeded the IAEA Safety Standards Series No. RS-G-1.7 screening criterion (sum of ratios ≥ 1) for naturally occurring radioactive materials, with total activities ranging from 74 to 12,992 Bq/kg. The highest residential concentration of 12,992 Bq/kg was detected at 5 m from the nearest workshop.

### Analysis details

#### Spatial distribution analysis.

The relationship between total radioactivity and distance is shown in Fig 3. No significant monotonic association was observed between distance from the nearest workshop and the residence-level representative total activity concentration (Spearman’s *ρ* = −0.122, *p* = 0.608). Using the median-distance threshold, the near-range group comprised 11 residential locations at distances of 7 m or less, and the far-range group comprised nine locations at distances greater than 7 m. The median total activities were 0.982 and 1.849 kBq kg^−1^ in the near- and far-range groups, respectively, and the corresponding standard deviations were 3.74 and 2.31 kBq kg^−1^. Neither the activity distributions (Mann–Whitney *U* = 45, *p* = 0.761) nor their dispersions (Brown–Forsythe *F* = 0.049, *p* = 0.828) differed significantly between the groups.

**Fig 3:**
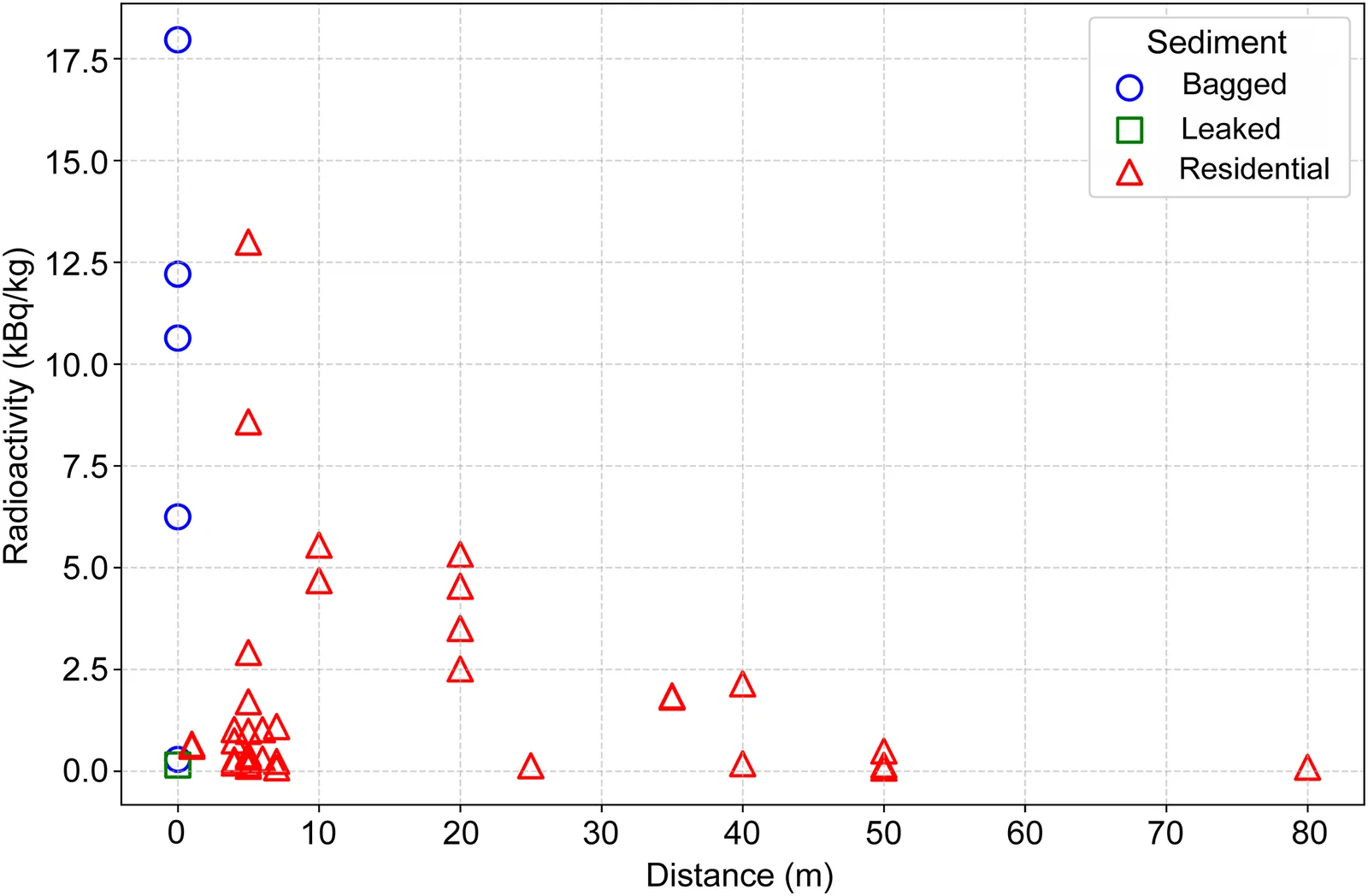
Total radioactivity as a function of distance from the nearest workshop. Horizontal axis, horizontal distance to the nearest workshop (m), with workshop samples plotted at 0 m; vertical axis, total radioactivity (Bq kg^−1^) as defined in Table 1. Points are categorised as bagged sediment (within workshops), leaked sediment (at workshop boundaries), and residential soil. Each of the 20 residential locations contributes one value, the higher of the totals at 10 and 20 cm, so that paired depth samples are not treated as independent.

The 1.5 × interquartile-range analysis identified two upper outliers: Residence 1, with a representative total activity of 12,992 Bq kg^−1^ at 5 m from the nearest workshop, and Residence 8, with 5,552 Bq kg^−1^ at 10 m. The larger descriptive standard deviation in the near-range group was strongly influenced by the extreme value at Residence 1 rather than by a general increase in activity variability across all near-range locations.

#### Depth distribution analysis.

Fig 4 shows the difference in total radioactivity between the 10 and 20 cm depths. Total activity was quantifiable at both depths for 17 residential locations: activity was higher at 10 cm at 11 locations, higher at 20 cm at five locations, and equal at one location. The median paired difference between the 10- and 20-cm samples was 82 Bq kg^−1^, with an interquartile range of −50–858 Bq kg^−1^, and the paired difference was not statistically significant (two-sided Wilcoxon signed-rank test, *n* = 16 after excluding one tied pair, *W* = 34, *p* = 0.079). Thus, the data did not demonstrate a consistent directional change in activity with depth. Nevertheless, the five locations that exhibited higher radioactivity at 20 cm than at 10 cm (Residence IDs 6, 8, 9, 10, and 19; negative differences in Fig 4), with absolute differences ranging from 50 to 1,035 Bq kg^−1^, indicate that elevated activity was not invariably confined to the shallower sampled layer.

**Fig 4:**
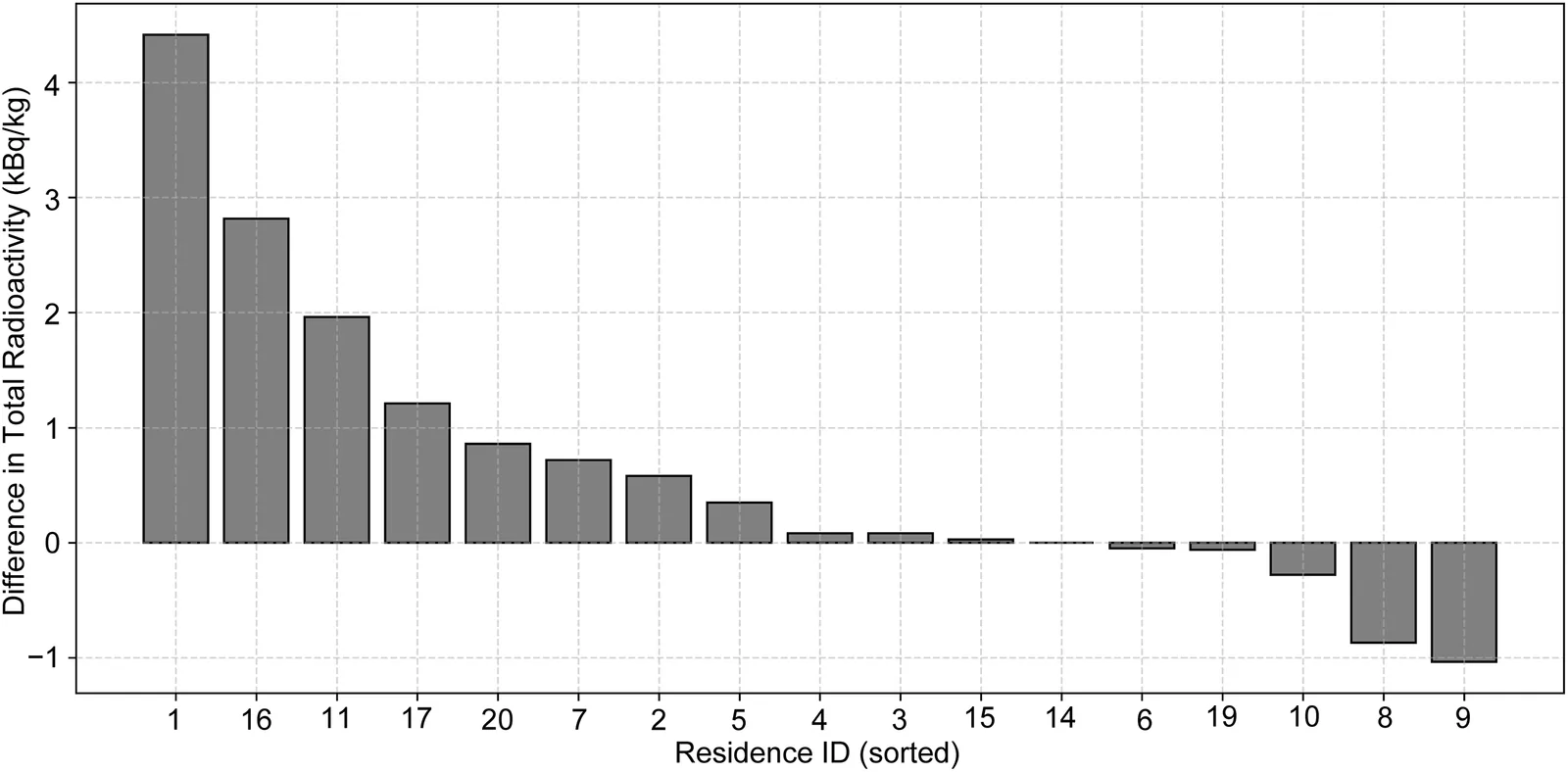
Difference in total radioactivity between the 10 and 20 cm depths at each residential location. Plotted values are total radioactivity at 10 cm minus that at 20 cm (Bq kg^−1^), with total radioactivity as defined in Table 1; positive values indicate higher activity at 10 cm. Only the 17 locations quantifiable at both depths are shown; Residences 12, 13, and 18 are excluded because one depth returned — (Table 1). Residence IDs are ordered by magnitude of the difference. Significance was assessed with a two-sided Wilcoxon signed-rank test on the 16 non-tied pairs (*W* = 34, *p* = 0.079).

#### Nuclide composition trends.

The relationships between the activity concentrations of each nuclide and total radioactivity (Bq/kg) are shown in Fig 5. The activity concentrations of the ^238^U and ^232^Th series increased approximately linearly with increasing total radioactivity, whereas ^40^K showed a weaker but positive correlation. The strength of these empirical relationships was summarized using descriptive R² values, which were 0.95 for ^238^U, 0.98 for ^232^Th, and 0.68 for ^40^K.

**Fig 5:**
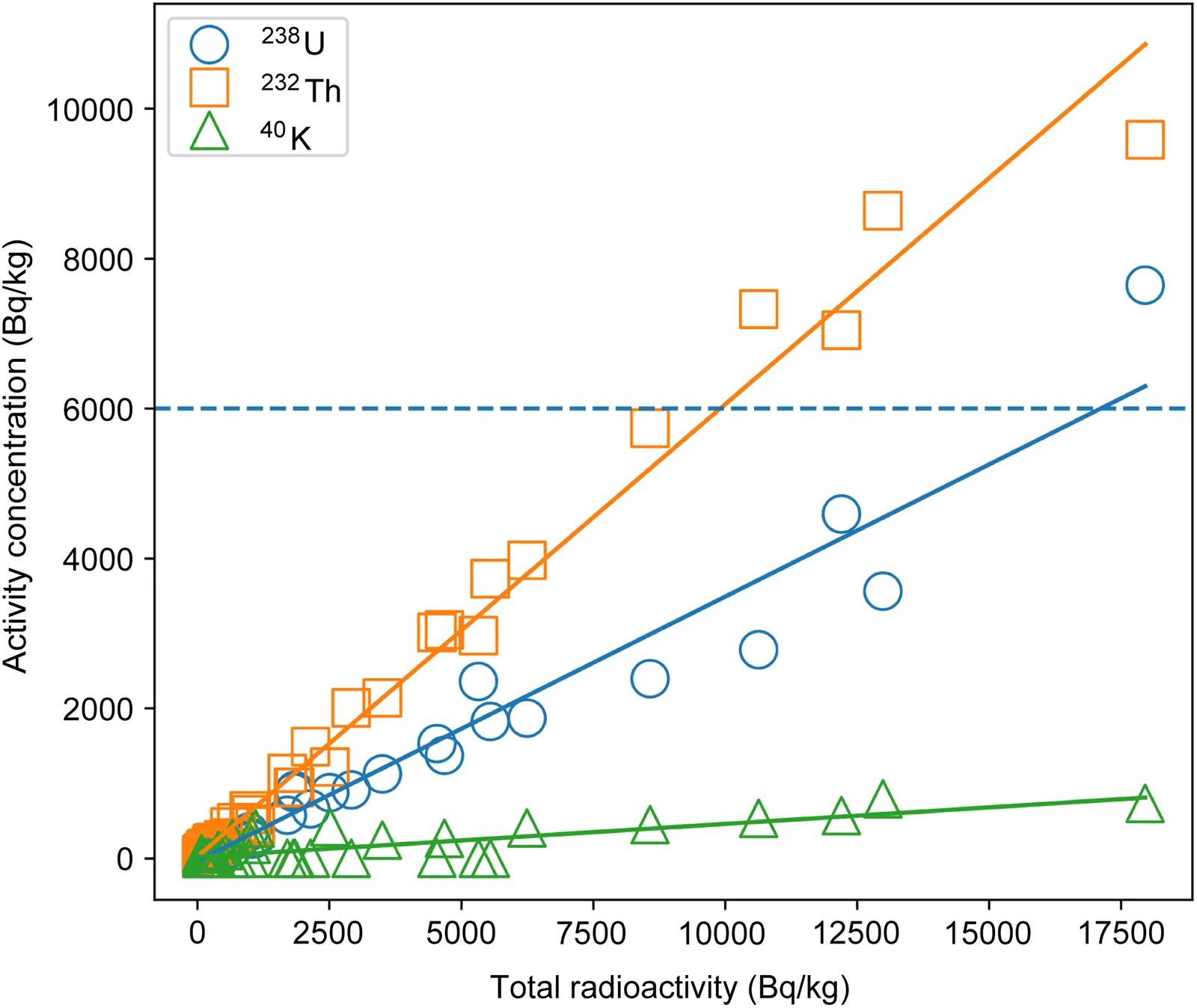
Activity concentrations of the ^238^U series, ^232^Th series, and ^40^K plotted against total radioactivity. Each point is one sample; both axes in Bq kg^−1^. The 43 of 46 samples with quantified totals are plotted (Sample IDs 30, 32, and 42 excluded; Table 1). Not-detected ^40^K values are plotted as zero, as in Table 1. Solid lines are ordinary least-squares regressions, and the reported R² values are descriptive rather than inferential (0.95, 0.98, and 0.68 for the ^238^U series, ^232^Th series, and ^40^K). The dashed line at 6,000 Bq kg^−1^ marks the lower bound of the ^238^U range reported by the IAEA for high-purity monazite sand.

#### Radiological screening indices.

Radiological screening indices (Equations 2–7) could be calculated for 43 of the 46 samples, including 37 of the 40 residential soil samples. Among the residential samples, the absorbed gamma dose rate *D* ranged from 41.3 to 6,895 nGy h^−1^ (median 343 nGy h^−1^), and the corresponding outdoor annual effective dose AEDE_out_ ranged from 0.051 to 8.46 mSv y^−1^ (median 0.42 mSv y^−1^). Twenty-four of the 37 samples exceeded the conventional screening reference value of 370 Bq kg^−1^ for Ra_eq_, and 24 exceeded *H*_ex_ = 1.

The highest screening estimates among the residential samples were obtained for Sample 7, collected at a depth of 10 cm at a residential location 5 m from Workshop 2, yielding Ra_eq_ = 1.60 × 10⁴ Bq kg^−1^, *D* = 6,895 nGy h^−1^, AEDE_out_ = 8.46 mSv y^−1^, *H*_ex_ = 43.1, *H*_in_ = 52.8, and an ELCR screening value of 3.3 × 10^−2^.

Thirteen of the 37 calculable residential samples yielded screening-level outdoor annual effective-dose estimates above 1 mSv y^−1^ under the generic occupancy assumptions used in the calculation, and seven of the 20 residential locations had at least one depth-specific screening estimate above 1 mSv y^−1^. These estimates do not constitute a regulatory compliance assessment or measurements of individual resident dose. Full sample-level results, including reference calculations for the workshop (bagged and leaked) samples, are presented in S1 Table.

## Discussion

Radioactivity levels in residential sediments showed pronounced spatial heterogeneity. The inferential analyses did not support a monotonic decrease in total activity with increasing distance from the nearest workshop, nor a significant difference in the distributions or dispersions of the near- and far-range groups; instead, the distribution was characterized by two localized extreme observations at 5 and 10 m from the nearest workshops, consistent with site-specific hotspots. Field observations confirmed that residues stored in torn plastic bags and material handling adjacent to residential buildings can promote leakage and scattering around workshop compounds (Fig 1a, b). The maximum residential concentration (12,992 Bq/kg) was detected within 5 m of a facility, and 32.5% of residential samples exceeded the IAEA RS-G-1.7 screening criterion. Comparable patterns of environmental dispersion have been reported in Nigeria’s Jos Plateau, where external dose rates of 10–80 μSv/h were recorded around processing facilities [6]. The radiological hazards documented here are also consistent with those recently reported for the amang (tin-tailing) processing industry in Peninsular Malaysia, where the same heavy-mineral assemblage is processed [23]. These findings demonstrate that small-scale mineral-processing activities can elevate contamination levels together with strong local variability in residential environments.

In interpreting these exceedances, it is important to note the regulatory meaning of the screening criterion. The value of 1 Bq/g for individual ^238^U- and ^232^Th-series radionuclides in IAEA Safety Guide RS-G-1.7 [22] is a screening level for exclusion, exemption, and clearance: materials below this level can generally be excluded from regulatory control, whereas exceedance does not automatically mandate remediation. Instead, it indicates that a graded, site-specific regulatory assessment is warranted, which may include periodic monitoring, controls on residue storage and reuse of contaminated soil, and restrictions on excavation or land-use change, proportionate to the assessed exposure. In the present context, the exceedance of the screening criteria in 13 of the 40 residential samples therefore identifies locations where such site-specific assessment and periodic monitoring are warranted.

The localized extreme observations near workshops are likely related to local redistribution processes in inhabited areas. Intense tropical rainfall can mobilize deposited particles and transport them along road surfaces and drainage pathways, and spillage during material handling and mechanical redistribution associated with vehicles may locally elevate concentrations near the source. However, these mechanisms were not measured directly in the present study, and the absence of statistically significant distance and depth effects should not be interpreted as evidence that workshop activities have no spatial influence; rather, given the limited number of residential locations, the results show that the contamination pattern is heterogeneous and cannot be represented adequately by a simple monotonic distance or depth model. Vertical profiles further indicate that contamination is not necessarily confined to the surface layer. Although the paired depth comparison showed no statistically significant systematic difference (Wilcoxon *p* = 0.079), five residential locations (Residence IDs 6, 8, 9, 10, and 19) exhibited higher total radioactivity at 20 cm than at 10 cm (negative differences in Fig 4). This inversion is consistent with earlier contamination that was subsequently covered by cleaner surface fill, leaving deeper layers with elevated concentrations despite lower surface values. Although the present dataset cannot distinguish burial from other subsurface redistribution processes, the results reflect vertical heterogeneity and suggest that surface-only measurements may underestimate deeper inventories relevant to long-term disturbance, such as excavation, gardening, and construction.

Nuclide composition patterns (Fig 5) provide additional insights into the source materials and mixing processes. Samples with very high ^238^U and ^232^Th activities (e.g., Sample IDs 3 and 4) approached the lower bound of activity concentrations reported by the IAEA for high-purity monazite sand, suggesting that heavy minerals containing monazite were present at high concentrations as a result of mineral processing activities at the workshops. Recent grade-resolved measurements of mechanically separated monazite show activity concentrations increasing steeply with monazite content [24], supporting the interpretation that the highest-activity samples reflect variable degrees of monazite enrichment. In contrast, several residential samples with low total radioactivity (<1,000 Bq/kg) exhibited relatively higher ^40^K concentrations, which is consistent with background soil characteristics unaffected by processing. Intermediate levels (1,000–5,000 Bq/kg) may reflect dispersion and mixing of leaked mineral concentrates into surrounding soils, with activity ranges comparable to those reported for designated radioactive mining waste disposal contexts in Malaysia [7]. Variability in the ^238^U and ^232^Th concentrations within this range may reflect selective transport and deposition of heavy minerals, which is consistent with the high specific gravities of monazite and zircon [13,14]. The comparatively lower specific gravities of common K-bearing minerals such as K-feldspar (~2.6) and biotite (~3.1) may partly explain the weaker correlation observed for ^40^K.

The radiological screening assessment quantitatively supports concern regarding several localized residential contamination hotspots. For context, the median calculated absorbed dose rate for the residential samples (343 nGy h^−1^) was approximately 5.8 times the worldwide population-weighted outdoor average of 59 nGy h^−1^ reported by UNSCEAR [19], while the maximum residential estimate (6,895 nGy h^−1^) was approximately 117 times that value. These comparisons are intended as screening-level context and do not represent measurements of individual resident dose.

The screening assessment addresses only external gamma exposure; site-specific characterization would additionally require in situ dose-rate measurements and personal or area dosimetry, together with consideration of source geometry, burial attenuation, shielding, occupancy, and age-specific behavior.

Several internal pathways are also potentially relevant at this site, consistent with current guidance on NORM in industrial processes [25]. Given the ^232^Th-dominated monazite contamination, inhalation of thoron and radon and their progeny—for which elevated exhalation rates have been reported regionally [11]—is potentially important, particularly indoors. Inhalation of resuspended monazite-bearing dust [8], inadvertent soil ingestion by children, and food-chain transfer constitute further pathways—radon and dust being the dominant contributors to worker doses in the Malaysian amang industry [23]. Leaching into shallow groundwater and surface water cannot be excluded given the water-based separation process and drainage-mediated redistribution; radon in water has been identified as a relevant exposure route in comparable settings [26,27]. None of these internal pathways was measured in the present study, and quantifying them is an essential component of the future site-specific exposure assessment.

This study has certain limitations. Measurements represent a single time point and may not capture seasonal dynamics under the tropical rainfall regime in Indonesia. Depth sampling was limited to 10 and 20 cm, leaving deeper profiles and behavior near the groundwater table unresolved. Mineralogical analyses (e.g., X-ray diffraction) of the collected samples were not performed; the association between radioactivity and specific heavy-mineral phases was therefore inferred from nuclide composition patterns rather than measured directly. Furthermore, because the workshops were selected through a risk-oriented, non-probabilistic design, the results should not be extrapolated to the entire Selindung District without additional probabilistic sampling. In addition, a full site-specific exposure and dose assessment was beyond the scope of this study; therefore, future studies should quantify occupational and residential exposures, including potential internal exposure pathways from radon and thoron inhalation, dust inhalation [8], ingestion, and groundwater use, and consider age-specific activity patterns, particularly for children. Future surveys based on probabilistic sampling would also enable geostatistical mapping of the contamination distribution at the district scale. Despite these constraints, the present study demonstrates that TENORM-related contamination from small-scale processing can extend into residential areas with substantial spatial and vertical heterogeneity, underscoring the need for site-specific assessment consistent with screening criteria (IAEA RS-G-1.7).

Practical measures could reduce residential exposure in the interim: periodic monitoring of soils around workshops, replacement of torn flexible bags with covered and contained storage of concentrates and residues, physical separation between storage areas and residential access routes, and restriction of children’s access to workshop compounds.

## Conclusions

This study demonstrated substantial TENORM contamination in residential areas surrounding small-scale mineral-processing workshops on Bangka Island. A total of 32.5% of residential soil samples exceeded the IAEA Safety Standards Series No. RS-G-1.7 screening criteria, indicating potential radiological concerns that warrant further site-specific quantitative dose assessment and exposure-pathway investigation.

Residential contamination was not explained by a statistically significant monotonic distance gradient. Instead, localized high-activity observations occurred within 5–10 m of the nearest workshops, including the maximum residential concentration of 12,992 Bq/kg at 5 m, demonstrating that elevated radioactivity extends beyond workshop boundaries into inhabited environments. These findings indicate that workshop proximity may be associated with site-specific hotspots, while local transport, handling, and depositional conditions are also likely to influence the contamination pattern.

Vertical profiles showed no significant systematic difference between the 10- and 20-cm samples; however, five locations exhibited higher radioactivity at 20 cm than at 10 cm, demonstrating that contamination was not invariably confined to the shallower sampled layer. The nuclide composition analysis also showed clear compositional trends, with relatively strong empirical relationships for the ^238^U and ^232^Th activity concentrations (R² = 0.95 and 0.98, respectively), while ^40^K showed a weaker correlation with total radioactivity (R² = 0.68).

Overall, these findings demonstrate that small-scale mineral-processing activities can cause spatially and vertically heterogeneous radiological contamination in residential settings, underscoring the need for site-specific assessment and management consistent with international screening criteria.

## Supporting information

S1 Checklist Inclusivity in global research questionnaire. (DOCX)

S1 Table Radiological screening indices for the 43 samples with quantifiable ^238^U- and ^232^Th-series activities, with the underlying activity concentrations in machine-readable form. Ra_eq_ (Bq kg^−1^), *D* (nGy h^−1^), *H*_ex_ and *H*_in_ (dimensionless), AEDE_out_ (mSv y^−1^), and ELCR (dimensionless) were calculated with Equations (2)–(7), using the ^238^U-series activity as a proxy for ^226^Ra. Activity concentrations are identical to Table 1; not-detected ^40^K was treated as zero. Sample IDs 30, 32, and 42 are absent because a series activity could not be quantified. Workshop sample values are reference calculations and were not used in the residential interpretation. N/A, not applicable (Distance and Depth are undefined for workshop samples). These are screening estimates under generic occupancy assumptions, not measurements of individual dose or risk.
(XLSX)
